# The auxiliary subunit KCNE1 regulates KCNQ1 channel response to sustained calcium-dependent PKC activation

**DOI:** 10.1371/journal.pone.0237591

**Published:** 2020-08-24

**Authors:** Xiaorong Xu Parks, Haani Qudsi, Chen Braun, Coeli M. B. Lopes

**Affiliations:** Aab Cardiovascular Research Institute, Department of Medicine, University of Rochester School of Medicine and Dentistry, Rochester, NY, United States of America; University of Milan, ITALY

## Abstract

The slow cardiac delayed rectifier current (IKs) is formed by KCNQ1 and KCNE1 subunits and is one of the major repolarizing currents in the heart. Decrease of IKs currents either due to inherited mutations or pathological remodeling is associated with increased risk for cardiac arrhythmias and sudden death. Ca^2+^-dependent PKC isoforms (cPKC) are chronically activated in heart disease and diabetes. Recently, we found that sustained stimulation of the calcium-dependent PKCβII isoform leads to decrease in KCNQ1 subunit membrane localization and KCNQ1/KCNE1 channel activity, although the role of KCNE1 in this regulation was not explored. Here, we show that the auxiliary KCNE1 subunit expression is necessary for channel internalization. A mutation in a KCNE1 phosphorylation site (KCNE1(S102A)) abolished channel internalization in both heterologous expression systems and cardiomyocytes. Altogether, our results suggest that KCNE1(S102) phosphorylation by PKCβII leads to KCNQ1/KCNE1 channel internalization in response to sustained PKC stimulus, while leaving KCNQ1 homomeric channels in the membrane. This preferential internalization is expected to have strong impact on cardiac repolarization. Our results suggest that KCNE1(S102) is an important anti-arrhythmic drug target to prevent IKs pathological remodeling leading to cardiac arrhythmias.

## Introduction

Voltage-gated potassium channels are ubiquitous and are major regulators of electrical properties of excitable cells. These potassium channels are assembled by four alpha subunits, each containing six transmembrane regions. Members of the single transmembrane KCNE family frequently co-assemble with potassium channel alpha subunits to form the native channel. For the cardiac potassium channel IKs, the alpha subunit (KCNQ1) and the beta subunit (KCNE1) are necessary to form the native channel [1, 2]. The KCNE1 subunit increases single channel conductance, slows the time course of channel activation and shifts voltage dependence of channel activation to more positive voltages [3–5]. In addition, expression of the KCNE1 subunit was shown to affect channel regulation by protein kinase A [6] and increase channel sensitivity to PIP_2_ 100-fold over the KCNQ1 subunit expressed alone [7].

Extensive studies have linked calcium-dependent PKC (cPKC) activation to heart failure, hypertrophy and diabetes [8–12]. Acute cPKC activation has been shown to regulate cardiac channels, including IKs, in heterologous and native systems [13–19]. In addition, we have recently showed that prolonged cPKC stimulation, specifically the PKCβII isoform, leads to IKs channel internalization [20, 21]. However, the role of the KCNE1 subunit on the PKCβII-mediated inhibition of channel membrane localization was not explored.

KCNE family members (KCNE1-KCNE5) are expressed in the heart and can interact with KCNQ1 [22, 23], although KCNE1 is the most abundant subunit [4]. Mutations in both the KCNQ1 and the KCNE1 subunits that decrease channel function are linked to Long QT syndrome, a disease that causes increased susceptibility to cardiac arrhythmias and sudden death [24]. KCNE1 subunit expression was shown to increase in insulin treated cells [25] and in human cardiomyopathy [22]. Several studies suggested that KCNE1 resides between the voltage sensor domains of two KCNQ1 subunits at the pore domain of the channel with either a KCNQ1:KCNE1 4:4 [26–31], 4:2 ratio [32–34] or multiple stoichiometries [35].

Here we studied the role of the KCNE1 subunit in the regulation of the IKs channel by sustained cPKC stimulation and more specifically, PKCβII activation. We first show that expression of the KCNE1 subunit is necessary for the down regulation of membrane expression of the channel in response to GqPCR stimulation and cPKC, with homomeric KCNQ1 channels do not responding to the stimulus. We also show that a mutation in the KCNE1 subunit (KCNE1(S102A)) abolished channel regulation both in heterologous systems and cardiomyocytes. Our results suggest that the KCNE1 subunit is critical for PKCβII-mediated regulation of channel membrane localization, and preferential internalization of IKs complexes (KCNQ1/KCNE1). Leaving homomeric KCNQ1 complexes at the plasma membrane may strongly impact cardiac repolarization.

## Methods and materials

### Constructs and chemicals

The following plasmid constructs were used in current study: hKCNQ1, hKCNE1, HA-α_1A_ adrenergic receptor (α_1A_-AR), hKCNQ1-GFP, hKCNE1(S102A) and eGFP, all previously described [13, 20]; Constitutively active PKCβII (CA-PKCβII) and dominant negative PKCβII (DN-PKCβII) were gifts from Bernard Weinstein (Addgene, cat#16384 and 16385, respectively) [36]. Plasmid vector pcDNA3.1(+) was used as a transfection control. The following adenoviral constructs were used: hKCNQ1-GFP, hKCNE1 (Vector Biolabs) and hKCNE1(S102A) (VectorBuilder). Cell permeable TAT-conjugated cPKC activator peptide (KAC1-1, SVEIWD Cys–Cys TAT_47-57_) [37] and a control peptide containing the HIV-TAT sequence (C1, TAT_47-57_) [38] were gifts from KAI Pharmaceuticals (South San Francisco, CA) [13, 17, 39–41]. The cell permeable pseudo-RACK1 activator peptide (KKWKMRRNQFWIKIQRC-CSVEIWD, containing a disulfide bridge between 17–1, Tocris Bioscience) was also used. Peptides were diluted in water and directly applied to either the extracellular media or culture media for overnight treatment at 1 μM concentration for the indicated time. LY333531 was purchased from Cayman Chemical Company. Go6976 and the membrane marker Di-8-ANEPPS were both purchased from Abcam. Dorsomorphin dihydrochloride was purchased from Tocris (Cat# 3093). Di-8-ANEPPS was applied 10 min before imaging. All chemicals were purchased from Sigma Aldrich unless otherwise specified.

### Cell culture and transient expression

HEK293T cells (ATCC, CRL-3216, passage 10–50) cultured (5% CO_2_ at 37°C) in DMEM (Corning Cellgro, 15-013-CV) supplemented with 10% FBS and 1% GlutaMax (Cellutron Life Technologies) were used for all non-cardiomyocyte experiments. Cells were transiently transfected with FuGene HD (Promega) following manufacturer’s instructions with the plasmid DNA of interest. Equal amounts of KCNQ1 (or KCNQ1-GFP) and KCNE1 plasmid DNA were used unless otherwise specified. Confocal and patch clamp experiments were performed 48–56 hours after transient expression.

### Adult cardiomyocyte isolation

Animal were cared at the University of Rochester vivarium. Cardiomyocyte isolation procedures were approved by the UCAR (University Committee on Animal Resources) guidelines at University of Rochester. Adult female rat hearts were used because of availability, to our knowledge, there are no known sex differences that would affect channel regulation. Ventricular cardiomyocytes were isolated. Briefly, heart was removed and placed in ice-cold Control Solution (mM: 133.5 NaCl, 4 KCl, 1.2 NaH₂PO₄, 10 HEPES, 1.2 MgSO₄, 11 Glucose. pH value was adjusted to 7.4 with NaOH,). Aorta was cannulated, and placed on a Langendorff perfusion apparatus. All perfused solutions were oxygenated (95% O_2_) and maintained at 37°C. Hearts were first perfused with calcium-containing solution for 5 min, followed by calcium-free solution with added 0.1% BSA for 5 min and calcium free solution with added collagenase (0.09% w/v) for 20–30 minutes until heart tissue was soft to touch. The ventricles were removed and minced to approximately 1mm pieces. Cells were than mechanically isolated using glass Pasteur pipettes cut to increasing diameters and collected by gravity. Calcium was progressively increased (μM: 50, 100, 200, 500 and 1000 CaCl_2_)_._ After isolation, cells were plated in coverslips coated with laminin for 2h (37°C, 5% CO_2_). Cells were then infected with adenovirus (AV-KCNQ1-GFP and AV-KCNE1 or AV-KCNE1(S102A) (1:1 KCNQ1:KCNE1 ratio) (1x10^8^ p.f.u./ml), overnight in 1ml of culture media (M-199) supplemented with 1–2% penicillin/streptomycin_._ Media was exchanged daily. Confocal and patch clamp experiments were performed 40–48 hours after infection.

### Cell treatment

Cells were treated with either 1 μM cPKC activator peptide or 30 μM Phenylephrine (Phe) for 90 min at 37°C in the extracellular recording solution (for details see solutions). After treatment, confocal images and patch clamp experiments were performed at room temperature.

### Confocal microscopy imaging

**Fig 1:**
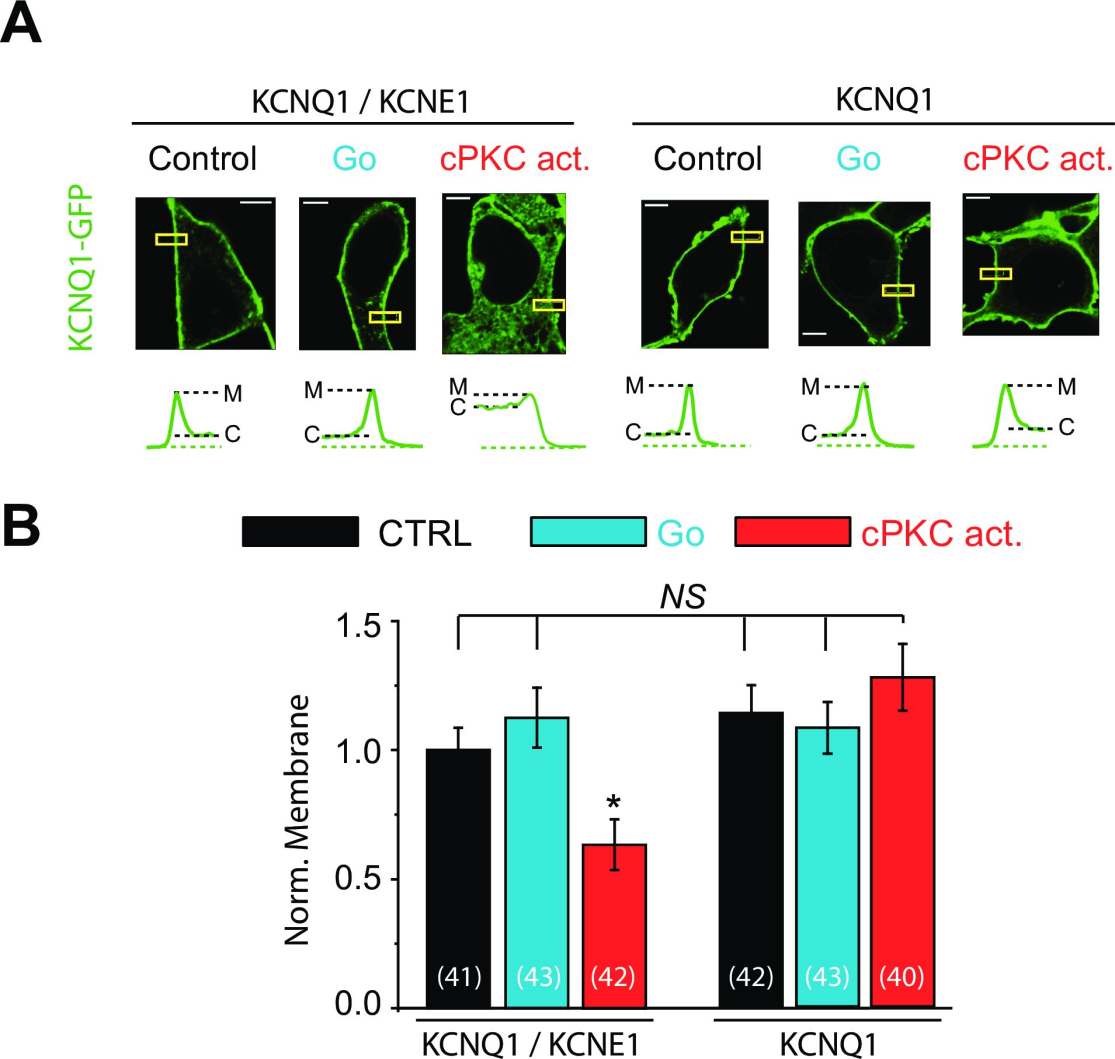
KCNE1 subunit is critical for KCNQ1 channel internalization. (A) *Top*, Representative confocal images of live HEK cells expressing KCNQ1-GFP with or without KCNE1 subunit after sustained cPKC activation (1 μM, cPKC activator pseudo RACK1, 90 min), and cPKC inhibition (1 μM, Go6976, 90 min). The same amount of vector pcDNA3.1(+) plasmid was used in place of KCNE1 in experiments of KCNQ1 alone. *Bottom*, the fluorescence profiles of the yellow boxed areas in images in top panels. M: cytoplasmic membrane; C: cytoplasm. The dotted green line marks the background fluorescence. (B) Summary data of normalized membrane localization of KCNQ1 in experiments conducted above. Scale bars, 5 μm. Control = vehicle. * p<0.05, (n = the number of cells).

For HEK cell experiments, cells were transfected with GFP-tagged KCNQ1 (1.5 μg), KCNE1 or KCNE1(S102A) (1.5 μg). For Phe experiments, cells were co-transfected with α_1A_-AR (3 μg). After transfection (6h), cells were plated on glass bottom dishes (MatTek Corporation). Before experiments cells were washed two times with PBS without calcium or magnesium (Quality Biological) and incubated at 37°C for treatment as above specified. Confocal images were taken with a confocal microscope (FV1000 Olympus, lenses: 60X oil) and analyzed with ImageJ software. KCNQ1-GFP fluorescence was measured at both plasma membrane (M) and cytosolic (C) regions by subtracting the background in randomly selected rectangular regions for each individual cells (see Fig 1). Membrane localization was calculated as the ratio of membrane to cytoplasm fluorescence (M/C) [20, 21, 42]. The average membrane localization obtained in control conditions for each experimental day was used for normalization of each individual experiment [20]. For cardiomyocytes, confocal data were quantified in a rectangular area perpendicular to the cell membrane. Rectangular selection was done avoiding the T-tubules.

### Immunochemistry

Cells were transfected with KCNQ1 (1.5 μg) and KCNE1-FLAG (1.5 μg). 6h after transfection, cells were split on 12mm cover slip. Forty-eight hours after transfection, cell are treated with cPKC activator for 90 min. After treatment, cells are washed with PBS and fixed with ice cold 4% PFA. Cells are blocked with 5% BSA PBS for 1h at room temperature. The primary antibody monoclonal mouse anti-Flag M2 antibody (Simga-Aldrich, catalogue # F1804) is added to the cell in a 1% BSA-PBS solution for 2.5h at room temperature. After 3 washes, the secondary antibody was added to the cells for 1h at room temperature. Fluorescence of KCNE1-FLAG was imaged using a confocal microscope, and the membrane localization of KCNE1-FLAG was calculated using ImageJ software in the same way as for KCNQ1-GFP. For immunoblotting: Cells were transfected with GFP-tagged KCNQ1 (1.5 μg) and KCNE1 (1.5 μg). Cells were harvested with Laemmli buffer supplemented with phosphatase and protease inhibitor (Thermo Fisher Scientific). Samples were sonicated, centrifuged for 2 min at 15,000g and supernatants were harvested as whole cell lysate. The samples were run on a 7% Acrylamide gel or with 4–20% Precast Protein Gels (Bio-Rad) under 95V for 2h. Anti-KCNE1 antibody (Santa Cruz technology sc-16796) and GAPDH (Santa Cruz technology sc-48166) were used together with donkey anti-goat IgG or donkey anti-rabbit IgG antibodies (LI-COR Biosciences). KCNE1 membrane fluorescence was measured using LI-COR (LI-COR Biosciences) and analyzed with ImageJ software.

### Electrophysiological data acquisition and analysis

HEK cells were transfected with KCNQ1 (0.5 μg) and either KCNE1 or KCNE1(S102A) (0.5 μg) plasmid, together with EGFP (0.1 μg) as a marker for transfected cells. Conventional whole-cell patch-clamp was performed 48–56 hours after transfection.

The extracellular recording solution contained (in mM): 145 NaCl, 5.4 KCl, 1.8 CaCl_2_, 1 MgCl_2_, 10 HEPES, 10 D-Glucose, pH adjusted to 7.4 with NaOH. The pipette solution contained (in mM): 130 K Aspartate, 11 EGTA, 1 MgCl_2_, 1 CaCl_2_, 10 HEPES, and 5 K_2_ATP, pH adjusted to 7.2 with KOH.

Cells were measured from -80 mV to +100 mV (3 seconds steps, 10 mV intervals), followed by a step to -20 mV. Axopatch 200B (Axon Instruments) was used for data acquisition and current data were analyzed using Clampfit software. Peak tail currents were fit with a Boltzmann equation, G(v) = Gmax/[1 + exp(-(V—V_1/2_)/*k*)] to calculate maximal conductance (Gmax) and half-maximal activation voltage (V_1/2_) as previously described [13].

### Statistical analysis

Results are shown as mean ± standard error of the mean. T-test was used for comparison of two experimental groups. For more than two independent group comparison, One-way ANOVA, followed by Dunnet’s test was used. The significance level was set at P<0.05.

## Results

### Expression of KCNE1 is necessary for cPKC-mediated channel internalization

We have recently reported that chronic stimulation of cPKC reduced the membrane localization of KCNQ1/KCNE1 channels and decreased channel function [20]. In order to study the role of KCNE1 subunit on cPKC-induced internalization of channels, we investigated the effect of chronic stimulation of cPKC on channel membrane localization in cells expressing KCNQ1-GFP either alone or together with the auxiliary subunit KCNE1. Because cPKC activation does not inhibit KCNQ1/KCNE1 membrane localization acutely, but over the course of one to two hours [20], we performed our experiments after 90min cPKC stimulation. Cells were stimulated with the specific cPKC activator peptide (cPKC act, 1 μM) for 90 min at 37°C. Membrane localization of KCNQ1 was measured using confocal microscopy. The plasma membrane (M) and cytoplasmic (C) fluorescence of KCNQ1-GFP were measured, and membrane localization was evaluated. Before stimulation, KCNQ1-GFP (in green) is strongly localized at the plasma membrane regardless of the expression of KCNE1 subunits (Fig 1A). Consistent with our previous work [20], membrane localization of KCNQ1-GFP in cells co-expressing KCNE1 was strongly inhibited after prolonged cell stimulation with the cPKC activator (Fig 1A). In the absence of KCNE1 expression, the channel remained in the membrane after cPKC stimulation (Fig 1A). We have previously shown that channel internalization in response to GqPCR stimulation was prevented by cPKC and PKCbetaII inhibitors in cells expressing KCNQ1 and KCNE1 subunits [20, 21]. In cells expressing only the KCNQ1-GFP subunit and α_1A_ adrenergic receptor, the channel remained in the membrane after treatment with the α_1A_-AR agonist phenylephrine for 90 min (Phe, 30 μM, S1 Fig), consistent with the lack of effect of cPKC observed (Fig 1).

To further investigate whether cPKC activation preferentially internalized heterometric channels we studied the effect of the cPKC activation on channel function. KCNE1 expression strongly regulates channel activation kinetics, shifting the voltage dependence of activation to more positive voltages, slowing kinetics of activation and increasing single channel conductance [1–5] (S2 Fig). To investigate the change of KCNQ1 homomeric contribution in response to chronic cPKC activation we focused on a voltage range where KCNQ1 homomeric channels show larger relative contribution, despite their lower conductance. We compared current elicited after 0.25 sec (largely the KCNQ1 component) and the additional current elicited between 0.25 and 2.75 sec (largely the KCNQ1/KCNE1 component) after depolarization. Our result shows that after cPKC activation there was a significant decrease on the late current component compared to the early current component, consistent with an enrichment of KCNQ1 homomers at the membrane (S2 Fig). Our data suggest that there is an increase in the contribution of the homomeric KCNQ1 current after cPKC activation.

Because of the very high membrane localization of the KCNQ1 channels in cells expressing KCNQ1 alone, in order to further investigate effects of cPKC on channel localization, we also studied changes of cytoplasmic channel expression, which may be more sensitive to cPKC treatment. Consistent with our previous results, the cytoplasmic KCNQ1 expression was significantly increased after cPKC stimulation in the presence of KCNE1 subunit [20]. In the absence of KCNE1, cytoplasmic KCNQ1 fluorescence was not significantly changed after treatment with the cPKC activator (S3 Fig).

### The membrane localization of KCNE1 subunit is decreased in response to cPKC activation

Although our previous work had shown internalization of the KCNQ1 subunit in response to sustained cPKC activation when KCNE1 subunits were expressed, we did not explore whether the KCNE1 subunit was internalized together with KCNQ1. To investigate whether KCNE1 subunits membrane expression was affected by sustained cPKC stimulation, we used FLAG-tagged KCNE1 subunits expressed together with KCNQ1 and labeled the cells with anti-FLAG antibodies in cells treated with a cPKC activator peptide (cPKC act, 1 μM for 90 min). Our data showed that membrane localization of KCNE1 subunits decreased in response to sustained cPKC activation (S4 Fig). To ascertain whether the overall expression of the KCNE1 subunit was affected by cPKC activator peptide treatment (cPKC act, 1 μM for 90 min), we quantified KCNE1 expression from whole cell extracts by Western Blots and showed no effect of cPKC activation on KCNE1 expression (S4 and S5 Figs for raw Western Blot).

### KCNE1 stoichiometry did not affect channel response to cPKC activation

**Fig 2:**
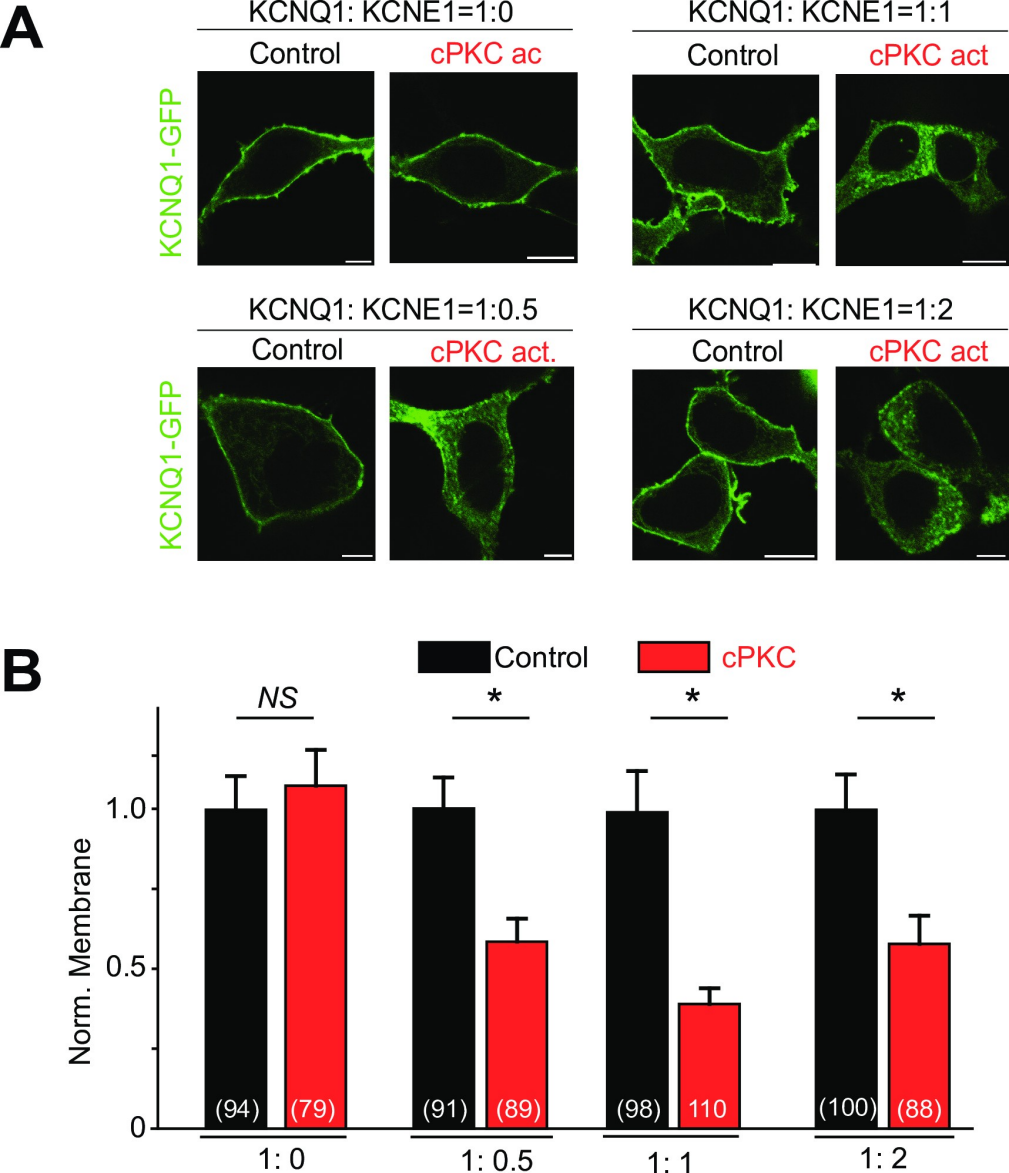
KCNQ1/KCNE1 channels internalized after sustained cPKC stimulation regardless of their stoichiometry. (A), Representative confocal images of HEK cells expressing KCNQ1-GFP and KCNE1 at different ratios as indicated, under sustained cPKC stimulation (1 μM cPKC activator peptide KAC1-1, 90 min). (B), Summary data of normalized KCNQ1-GFP membrane localization. Control = control peptide C1. Scale bars, 5 μm. *p<0.05, (n = number of cells).

Because multiple stoichiometries between KCNQ1 and KCNE1 have been suggested [35], we investigate the role of KCNQ1:KCNE1 stoichiometry on channel regulation by sustained cPKC activation. In cells expressing a constant amount of the KCNQ1 subunit, we co-expressed increasing amounts of the KCNE1 subunit (KCNQ1:KCNE1 ratios of 1:0, 1:0.5, 1:1 and 1:2). For all the KCNQ1:KCNE1 ratios tested, the membrane localization of KCNQ1-GFP subunits decreased in response to cPKC activation, as long as KCNE1 subunits were expressed. Our results suggest that the presence of KCNE1 subunits, even at low expression levels, confers sensitivity to sustained cPKC activation (Fig 2).

### KCNE1(S102A) mutation prevents PKCβII-dependent IKs channel internalization

**Fig 3:**
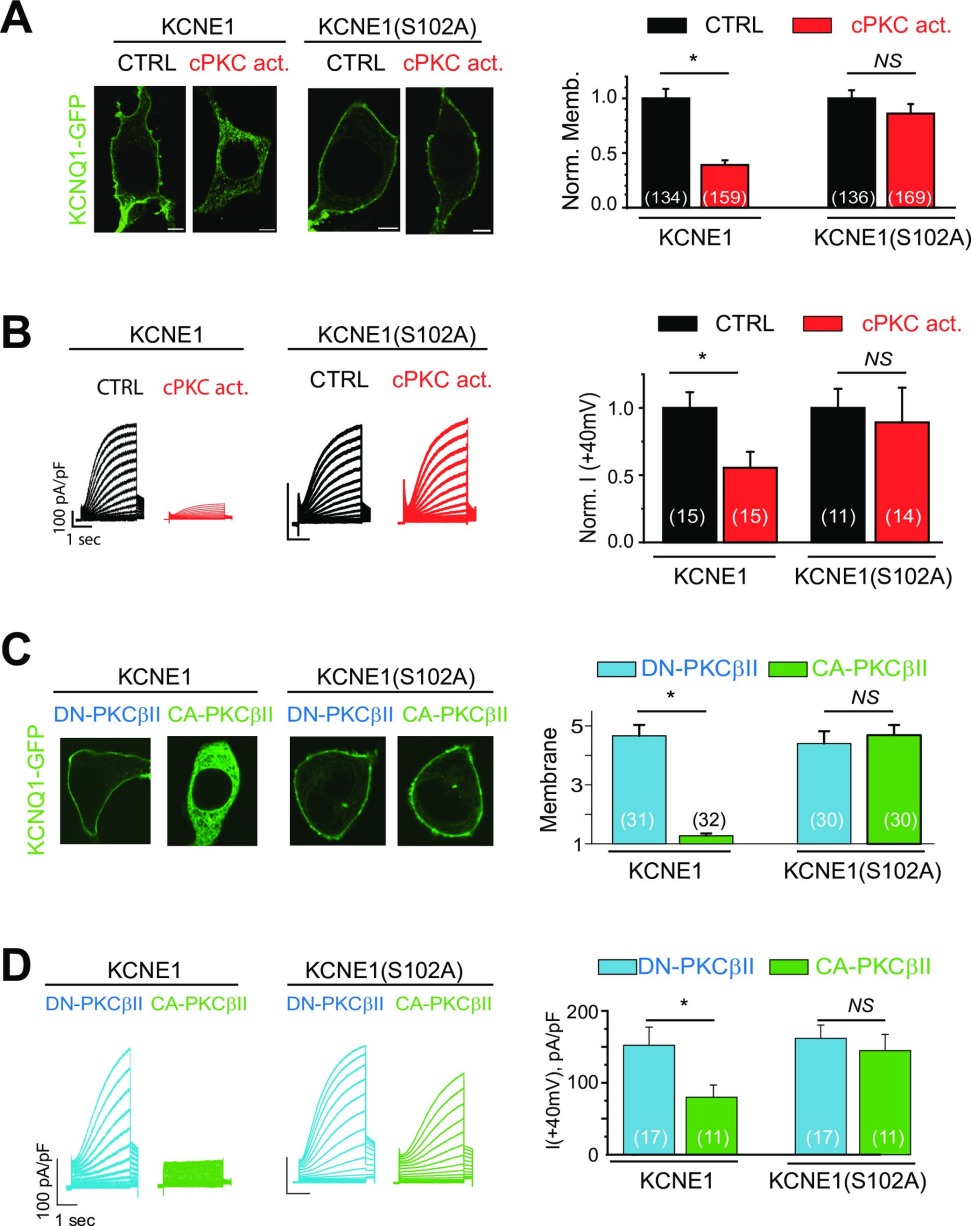
Serine 102 of KCNE1 is involved in IKs internalization after cPKC stimulation. (A) *Left*, Representative confocal images of HEK cell expressing KCNQ1-GFP and KCNE1 or KCNE1(S102A) and after sustained cPKC stimulation (cPKC activator peptide KAC1-1, 1 μM, 90 min). *Right*, Summary data of normalized KCNQ1 membrane localization. (B) *Left*, Representative IKs current traces of HEK cells expressing KCNQ1 and KCNE1 or KCNQ1 and KCNE1(S102A) after cPKC stimulation (cPKC activator peptide KAC1-1, 1 μM, 90 min). *Right*, Summary data of normalized current density at +40 mV in experiments conducted to the left. (C) *Left*, Representative confocal images of HEK cells expressing KCNQ1-GFP and KCNE1 or KCNE1(S102A) together with DN-PKCβII or CA-PKCβII. *Right*, Summary data of KCNQ1 membrane localization. (D) *Left*, Representative IKs current traces of HEK cells expressing KCNQ1 and KCNE1 or KCNQ1 and KCNE1(S102A) together with DN-PKCβII or CA-PKCβII. *Right*, Summary data of current density at +40 mV in experiments conducted to the left. Control = control peptide C1 in Panel A and B. Scale bars, 5 μm. *p<0.05, (n = number of cells).

KCNE1(S102) has been shown to be important for channel acute regulation by cPKC [13, 43–46]. To investigate the role of KCNE1(S102) on regulation of the KCNQ1/KCNE1 channel membrane localization by prolonged cPKC stimulation, KCNQ1-GFP was expressed either together with KCNE1(WT) or with the KCNE1(S102A) mutant subunit. KCNQ1-GFP membrane localization in KCNQ1 and KCNE1(S102A) expressing cells was not different from the membrane localization of KCNQ1-GFP in KCNQ1 and KCNE1(WT) expressing cells. Nonetheless, KCNE1(S102A) expressing channels remained on the cell surface after sustained cPKC activation (Fig 3A). To investigate the role of KCNE1(S102) on PKCβII regulation of IKs channel membrane localization, either constitutively active PKCβII (CA-PKCβII) or dominant negative PKCβII (DN-PKCβII) together with KCNQ1-GFP and the KCNE1 subunits were expressed. When KCNE1(S102A) subunits were expressed, channels remained on the cell surface, despite expression of CA-PKCβII, while the membrane localization of KCNQ1/KCNE1(WT) channels was significantly reduced in the presence of CA-PKCβII (Fig 3C). These results suggest that PKCβII phosphorylation of KCNE1(S102) is necessary for KCNQ1/KCNE1 internalization.

To investigate the role of KCNE1(S102) to cPKC regulation of channel function, we first measured the effect of cPKC activation in cells expressing untagged KCNQ1 subunits co-expressed with either KCNE1(WT) or KCNE1(S102A) subunits. For cells expressing KCNE1(WT), currents were inhibited by the cPKC activator treatment, while KCNE1(S102A) expressing cells show no response to cPKC (Fig 3B). The unstimulated current was not significantly affected by KCNE1(S102A) mutation. As we previously shown for the cells expressing KCNE1(WT) subunit [20], the voltage dependence of activation was also not affected by cPKC treatment in cells expressing KCNQ1 and KCNE1(S102A) (S6 Fig). We also measured channel currents in cells expressing KCNQ1 and KCNE1 untagged subunits together with either CA-PKCβII or DN-PKCβII. Consistent with our previous data, currents were significantly decreased in CA-PKCβII expressing cells compared to DN-PKCβII for cells co-expressing KCNQ1 and KCNE1(WT) subunits [20, 21] (Fig 3D). For cells expressing the KCNE1(S102A) subunit, channel function was not significantly different between CA-PKCβII and DN-PKCβII expressing cells (Fig 3D). These results suggest that phosphorylation of KCNE1(S102) is necessary for regulation of channel function by PKCβII.

### KCNE1(S102A) mutation prevents cPKC-dependent IKs channel internalization in cardiomyocytes

**Fig 4:**
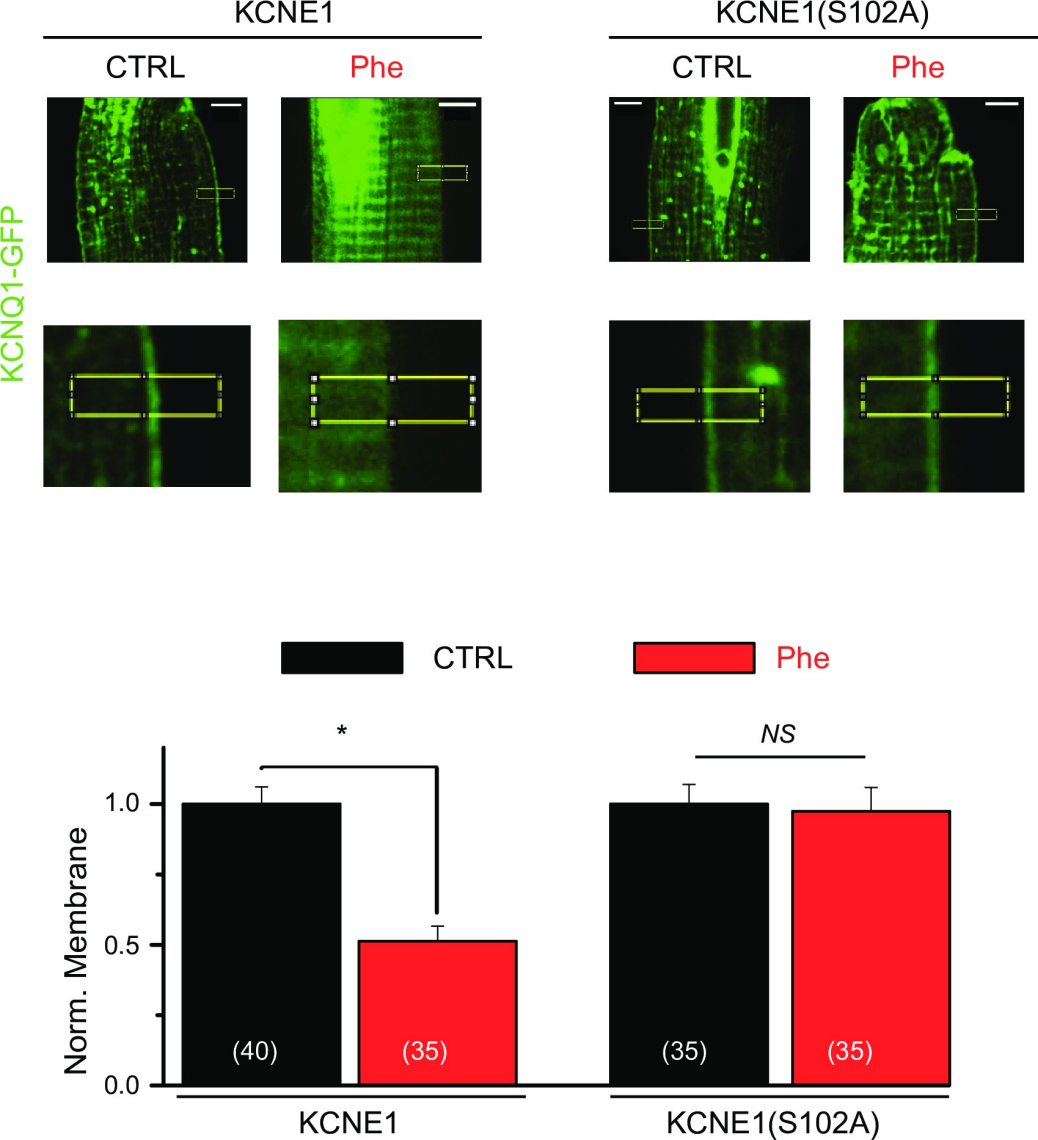
KCNE1(S102A) mutation prevents cPKC-dependent IKs channel internalization in isolated adult rat ventricular myocytes. *Top*, Representative images of cardiomyocytes expressing adenovirus of KCNQ1-GFP and KCNE1 or KCNE1(S102A), with sustained α1-AR stimulation (Phe, 30 μM, 90 min), and enlarged yellow boxed areas. *Bottom*, Summary data of normalized membrane localization of KCNQ1 from experiments conducted as in top panel. Control = vehicle. Scale bars, 5 μm. *p<0.05, n = number of cells measured.

To investigate the role of KCNE1(S102) on cPKC regulation of IKs channel membrane localization in the native system, KCNQ1-GFP was expressed with either wild-type KCNE1(WT) or KCNE1(S102A) in cardiomyocytes using adenoviral infection. We have previously shown that prolonged α1-AR activation inhibits channel membrane localization via cPKC activation, and inhibits KCNQ1/KCNE1 currents in cardiomyocytes [20, 21], although the role of KCNE1 was not explored. Thus, we treated cardiac myocytes with the α1-AR agonist phenylephrine (Phe, 30 μM) for 90 min. Consistent with our previous results [20], the WT channel membrane localization was strongly inhibited in response to Phe treatment (Fig 4). In the absence of stimulation, KCNQ1 and KCNE1(S102A) infected cardiomyocytes show the same membrane localization as the one measured for KCNQ1 and KCNE1(WT) cells. Nonetheless, KCNE1(S102A) expressing channels remained on the cell surface after agonist-induced cPKC activation (Fig 4), consistent with the results observed in HEK cells.

To confirm KCNQ1/KCNE1 channel regulation is independent from subunit stochiometry in cardiomyocytes, we also expressed human IKs channel subunits at an increased KCNQ1 ratio (KCNQ1:KCNE1, 3:1). Cardiac cells were stimulated with Phe (30 μM, 90 min). As for cells with the 1:1 infection ratio (Fig 4), KCNQ1-GFP localized at the sarcolemmal membrane in unstimulated cells (S7 Fig). KCNQ1:KCNE1 (3:1) infected cardiomyocytes showed a significant decrease in KCNQ1-GFP membrane expression in response to Phe, similar to the KCNQ1:KCNE1 (1:1) infected (compare Fig 4 to S7 Fig). Phe response of cells expressing KCNQ1-GFP and KCNE1 at 3:1 ratio was blocked by PKCβII inhibitors in a similar manner as that of 1:1 expression ratio, consistent with our previous results [20] (S7 Fig).

## Discussion

Here we found that homomeric KCNQ1 complexes are not regulated by sustained stimulation of calcium-dependent PKC isoforms. In addition we show that KCNE1(S102) phosphorylation site is critical in regulation of the channel membrane localization by calcium-dependent PKC isoforms. These findings provide a novel mechanism by which cardiomyocytes can preferentially internalize IKs complexes (KCNQ1/KCNE1), leaving homomeric KCNQ1 complexes at the plasma membrane. As KCNE1 is critical for KCNQ1 characteristic channel function [1–7], modulating surface expression to favor homomeric KCNQ1 channels could alter cardiomyocyte repolarization and therefore alter cardiac rhythm.

The C-terminus of KCNQ1 has been shown to interact with the C-terminus of KCNE1, and form a macromolecular complex with other molecules, including PI(4,5)P2, calmodulin, Nedd4.2 and AKAP-Yotiao, responsible for proper assembly, function and regulation of the channel [47–51]. KCNE1 expression has been shown to be important for regulation of the channel by PKA, PI(4,5)P2 and acute activation of the channel by PKC [6, 7, 13]. In addition, the Long QT associated mutation KCNE1(D76N) has been shown to decrease membrane expression in KCNQ1/KCNE1 channels in *Xenopus oocytes* [24], suggesting KCNE1 is important in channel regulation of membrane expression. KCNQ1 subunits alone have been recently suggested to internalize in response to Phe, although recycling and overall membrane expression were not measured in that study [52]. In addition, internalization of KCNQ1 in the presence and absence of KCNE1 was not compared quantitatively. Our data indicated that KCNE1 is a major regulator of channel internalization in response to α1-AR, but did not preclude a minor PKCβII-independent component of the α1-AR-mediated KCNQ1 internalization in the absence of KCNE1.

KCNQ1 subunits expressed alone have been shown to undergo dynamin-dependent internalization in response to AMP-activated protein kinase (AMPK) activation in kidney cells [53, 54]. PKCβII inhibition was shown to increase AMPK activity [55]. We do not observe changes in membrane localization of KCNQ1 in response to PKCβII inhibition either in the presence or absence of KCNE1 [20] (Fig 1), suggesting PKCβII-AMPK signaling is not an important mechanism in the cPKC-mediated internalization observed. This is consistent with the lack of cPKC effect observed when KCNQ1 subunits expressed alone. Although we observe an overall decrease in KCNQ1 plasma membrane localization in response to PKCβII activation, we also investigated whether in addition to KCNQ1-KCNE1(S102) mediated internalization, PKCβII activation leads to an increase of KCNQ1 membrane localization, possibly mediated by AMPK inhibition, contributing to an enrichment of KCNQ1 homomeric channels. We see no evidence for such mechanism, given cytoplasmic KCNQ1 expression was not significantly affected by cPKC activation in cells expressing KCNQ1 alone. Overall, our data show that KCNE1 is necessary for PKCβII regulation of the channel membrane localization.

KCNE1(S102) is a conserved putative PKC phosphorylation site in KCNE1. The equivalent residue to KCNE1(S102A) has been suggested to underlie the inhibition of mice and rat IKs in response to PKC [44]. KCNE1(S102) was also suggested to regulate the basal function and KCNQ1/KCNE1 membrane localization for a short KCNQ1 isoform with a truncated N-terminus [43, 56]. Our data show that the KCNE1(S102A) mutation does not increase channel current or membrane localization both in HEK cells and cardiomyocytes in the absence of cPKC stimulation (Figs 3 and 4). In contrast to our results, the KCNE1(S102A) mutation was shown to increase basal currents for channels formed by the short KCNQ1 variant when co-expressed with KCNE1 subunits in COS cells [56]. This variant may show different regulation than the fully functional channel. We have previously shown that acute cPKC activation leads to a negative shift of the channel voltage dependence due to phosphorylation of KCNE1(S102) in minutes [13]. This acute increase of channel function is followed by channel internalization over the course of one to two hours [20]. We here show that the same KCNE1(S102) residue that is responsible for the acute regulation also leads to channel internalization, suggesting that the channel phosphorylation leads to internalization as a feedback mechanism that limits prolonged channel activation. Consistently, we showed channel inhibition is observed only after washout of the cPKC stimulus [20]. Here we show that KCNE1 expression, and more specifically KCNE1(S102) phosphorylation, is critical to the decrease in membrane localization of the KCNQ1/KCNE1 channel in response to prolonged GqPCR stimulation and PKCβII activation. We show that KCNE1(S102A) does not change channel membrane localization in the absence of stimulus. Hence, the S102 site at the KCNE1 subunit provides a specific target for cardiac anti-arrhythmic drugs to prevent pathological remodeling in response to prolonged stress stimulus.

## Supporting information

S1 Fig Homomeric KCNQ1 channels remained on plasma membrane under sustained α1 adrenergic stimulation. *Left*, representative confocal images of HEK293T cells expressing KCNQ1 and α_1A_-AR, treated with Phe (30 μM for 90 min). *Right*, summary data of experiments conducted. Control = vehicle. Scale bars, 5 μm. n = number of cells tested.
(DOCX)

S2 Fig KCNQ1 homomeric-like current enriched after chronic cPKC activation in HEK cells expressing KCNQ1 and KCNE1. (A) Representative traces of current elicited from a +20mV depolarizing step from a holding potential of -80mV in HEK293T cells transiently expressing KCNQ1 alone (left), and KCNQ1 and KCNE1 in either the absence (middle) or presence (right) of cPKC activation (cPKC activator peptide KAC1-1, 1 μM, 90 min). Control cells were treated with control peptide (C1). The early current was measured 0.25 s after the depolarizing step (Iearly), and the late current was the current activated between 0.25 s and 2.75 s after the depolarizing step (Ilate), as indicated. Dashed lines marked the 0 current. (B), Summary data of the ratio of Ilate:Iearly for depolarizing steps from 0 to +40 mV. *p<0.05.
(DOCX)

S3 Fig Cytoplasmic KCNQ1 expression increased after chronic cPKC activation in cells expressing KCNQ1 and KCNE1. (A), Representative confocal images of HEK cells expressing KCNQ1-GFP in the presence and absence of KCNE1 subunit, in the presence and absence of cPKC activation (cPKC activator pseudo RACK1, 1 μM, 90 min). Cytoplasmic fluorescence was measured as indicated and normalized to background fluorescence (intracellular fluorescence-background fluorescence)/ (background fluorescence). (B), Summary data of KCNQ1 cytoplasmic fluorescence in experiments conducted as in the top panels. CTRL = vehicle. Scale bars, 5 μm. *p<0.05. (n = cell number).
(DOCX)

S4 Fig The membrane localization of KCNE1 subunits is decreased in response to sustained cPKC stimulation without change of overall subunit expression. (A) *Left*, Representative immunofluorescent images of fixed HEK cells expressing KCNQ1 and FLAG-KCNE1 after sustained cPKC stimulation (1 μM cPKC activator peptide KAC1-1, 90 min). Nuclei were labeled with DAPI (in blue). *Right*, Summary data of normalized KCNE1 membrane localization. (B) *Left*, Representative Western Blots of whole-cell extracts obtained after sustained cPKC activation (1 μM cPKC activator peptide KAC1-1, 90 min). UNT. was untransfected sample. Data were normalized to GAPDH loading control expression. *Right*, Average of KCNE1/GAPDH expression ratio in 3 experiments. Control = control peptide C1. Scale bars, 5 μm. *p<0.05, (n = number of cells).
(DOCX)

S5 Fig Western Blots of whole-cell extracts obtained after sustained cPKC activation. Western Blots of whole-cell extracts obtained after sustained cPKC activation (1 μM cPKC activator peptide KAC1-1, 90 min). UNT. marks untransfected samples.
(DOCX)

S6 Fig Sustained cPKC activation did not change voltage dependence of KCNQ1/KCNE1(S102A) channel activation. Average I-V plots (left) and V_1/2_ (right) of KCNQ1/KCNE1(S102A) channels at control condition (in black, control peptide C1) and sustained cPKC activation (1 μM cPKC activator peptide KAC1-1) for 90 min (in red). The solid lines in the left panels are Boltzman fits of experimental data. *p<0.05, n = the number of cells measured.
(DOCX)

S7 Fig Change in KCNQ1:KCNE1 stoichiometry does not affect cPKC-dependent IKs channel internalization in isolated adult rat ventricular myocytes. *Left*, Representative images of cardiomyocytes expressing adenovirus of KCNQ1-GFP and KCNE1 at a ratio of 3:1, in the presence and absence of sustained α1-AR stimulation (Phe, 30 μM, 90 min), and cPKC inhibition (20 nM, LY333531, 90 min). *Right*, Summary data of membrane localization of KCNQ1 from experiments conducted as in the left panel. *p<0.05, n = number of cells measured.
(DOCX)

S8 Fig AMPK inhibitor does not affect KCNQ1 membrane localization and cPKC mediated internalization. (A) Left: representative confocal images of HEK cells expressing KCNQ1-GFP and the KCNE1 subunit, in the presence and absence of dorsomorphine (90 min). Cytoplasmic fluorescence was measured at the indicated region. Right: Summary data of KCNQ1 normalized membrane localization in experiments conducted as in the left panels. (B) Top: representative confocal images of HEK cells expressing KCNQ1-GFP and KCNE1 treated with Phe (30uM, 90min) in the presence and absence of dorsomorphine (90 min). Bottom: Summary data of KCNQ1-GFP cytoplasmic fluorescence in experiments conducted as in the top panels for cells treated with either Phe (30uM, 90min) or cPKCact (1uM, 90min) as indicated. For all conditions, control is significantly different from treated cells. Scale bars, 5 μm. *p<0.05. (n = cell number).
(DOCX)

S1 Raw images (PDF)
