# Supplementary material for: The auxiliary subunit KCNE1 regulates KCNQ1 channel response to sustained calcium-dependent PKC activation
Source: PLoS One. 2020 Aug 24;15(8):e0237591. doi: 10.1371/journal.pone.0237591 (PMC7446858; doi:10.1371/journal.pone.0237591)
Supplement: S1 Fig — Left, representative confocal images of HEK293T cells expressing KCNQ1 and α1A-AR, treated with Phe (30 μM for 90 min). Right, summary data of experiments conducted. Control = vehicle. Scale bars, 5 μm. n = number of cells tested. (DOCX) [file pone.0237591.s001.docx]

**
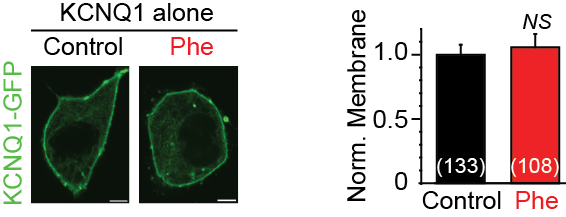
**

**Figure S1. Homomeric KCNQ1 channels remained on plasma membrane under sustained α1 adrenergic stimulation.** *Left*, representative confocal images of HEK293T cells expressing KCNQ1 and α_1A_-AR, treated with Phe (30 µM for 90 min). *Right*, summary data of experiments conducted. Control = vehicle. Scale bars, 5 µm. n = number of cells tested.
