# Supplementary material for: The auxiliary subunit KCNE1 regulates KCNQ1 channel response to sustained calcium-dependent PKC activation
Source: PLoS One. 2020 Aug 24;15(8):e0237591. doi: 10.1371/journal.pone.0237591 (PMC7446858; doi:10.1371/journal.pone.0237591)
Supplement: S2 Fig — (A) Representative traces of current elicited from a +20mV depolarizing step from a holding potential of -80mV in HEK293T cells transiently expressing KCNQ1 alone (left), and KCNQ1 and KCNE1 in either the absence (middle) or presence (right) of cPKC activation (cPKC activator peptide KAC1-1, 1 μM, 90 min). Control cells were treated with control peptide (C1). The early current was measured 0.25 s after the depolarizing step (Iearly), and the late current was the current activated between 0.25 s and 2.75 s after the depolarizing step (Ilate), as indicated. Dashed lines marked the 0 current. (B), Summary data of the ratio of Ilate:Iearly for depolarizing steps from 0 to +40 mV. *p<0.05. (DOCX) [file pone.0237591.s002.docx]

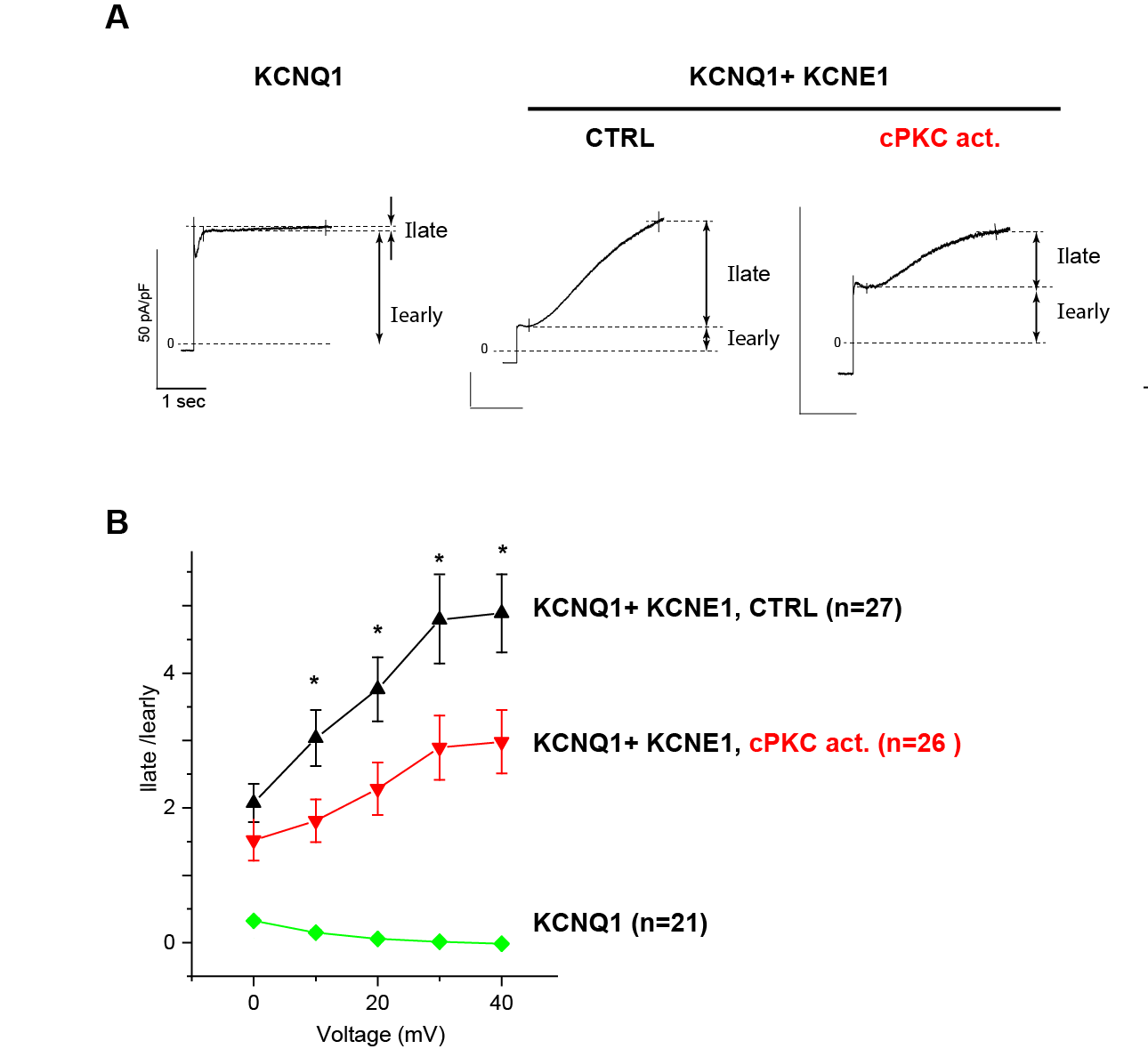


**Figure S2. KCNQ1 homomeric-like current enriched after chronic cPKC activation in HEK cells expressing KCNQ1 and KCNE1.**

**(A)** Representative traces of current elicited from a +20mV depolarizing step from a holding potential of -80mV in HEK293T cells transiently expressing KCNQ1 alone (left), and KCNQ1 and KCNE1 in either the absence (middle) or presence (right) of cPKC activation (cPKC activator peptide KAC1-1, 1 µM, 90 min). Control cells were treated with control peptide (C1). The early current was measured 0.25 s after the depolarizing step (Iearly), and the late current was the current activated between 0.25 s and 2.75 s after the depolarizing step (Ilate), as indicated. Dashed lines marked the 0 current. **(B),** Summary data of the ratio of Ilate:Iearly for depolarizing steps from 0 to +40 mV. *p<0.05.
