# Supplementary material for: The auxiliary subunit KCNE1 regulates KCNQ1 channel response to sustained calcium-dependent PKC activation
Source: PLoS One. 2020 Aug 24;15(8):e0237591. doi: 10.1371/journal.pone.0237591 (PMC7446858; doi:10.1371/journal.pone.0237591)
Supplement: S3 Fig — (A), Representative confocal images of HEK cells expressing KCNQ1-GFP in the presence and absence of KCNE1 subunit, in the presence and absence of cPKC activation (cPKC activator pseudo RACK1, 1 μM, 90 min). Cytoplasmic fluorescence was measured as indicated and normalized to background fluorescence (intracellular fluorescence-background fluorescence)/ (background fluorescence). (B), Summary data of KCNQ1 cytoplasmic fluorescence in experiments conducted as in the top panels. CTRL = vehicle. Scale bars, 5 μm. *p<0.05. (n = cell number). (DOCX) [file pone.0237591.s003.docx]

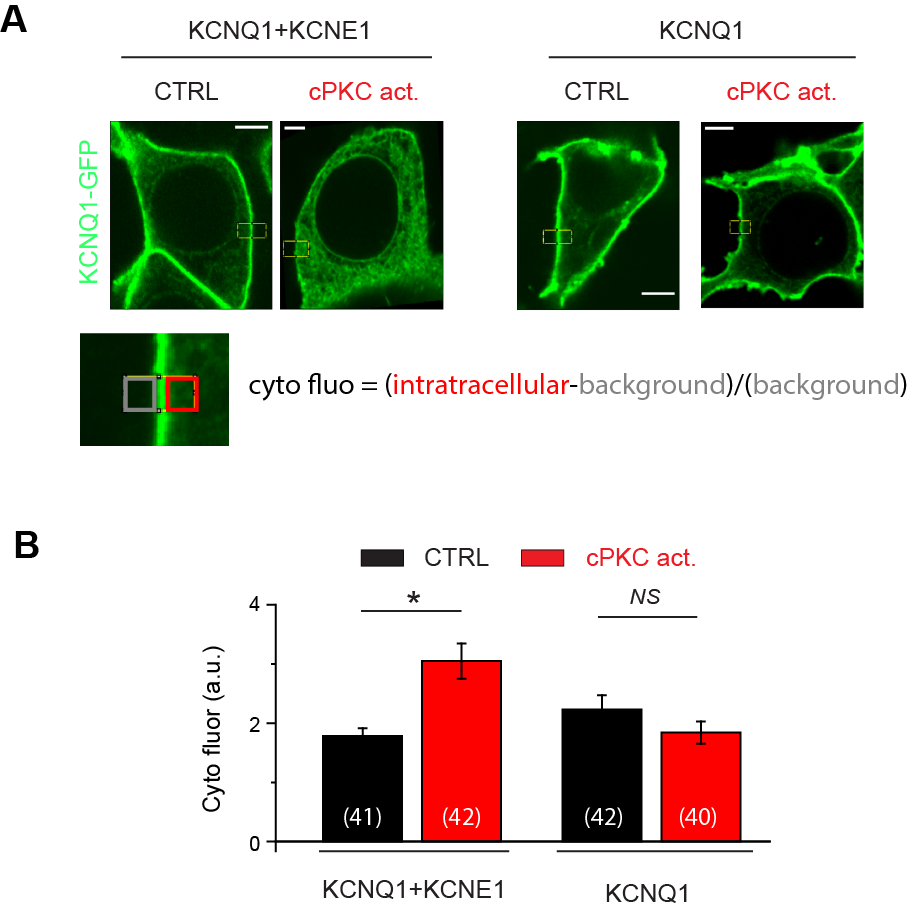


**Figure S3. Cytoplasmic KCNQ1 expression increased after chronic cPKC activation in cells expressing KCNQ1 and KCNE1.**

**(A),** Representative confocal images of HEK cells expressing KCNQ1-GFP in the presence and absence of KCNE1 subunit, in the presence and absence of cPKC activation (cPKC activator pseudo RACK1, 1 µM, 90 min). Cytoplasmic fluorescence was measured as indicated and normalized to background fluorescence (intracellular fluorescence-background fluorescence)/ (background fluorescence). **(B),** Summary data of KCNQ1 cytoplasmic fluorescence in experiments conducted as in the top panels. CTRL = vehicle. Scale bars, 5 µm. *p<0.05. (n = cell number.)
