# Supplementary material for: The auxiliary subunit KCNE1 regulates KCNQ1 channel response to sustained calcium-dependent PKC activation
Source: PLoS One. 2020 Aug 24;15(8):e0237591. doi: 10.1371/journal.pone.0237591 (PMC7446858; doi:10.1371/journal.pone.0237591)
Supplement: S4 Fig — (A) Left, Representative immunofluorescent images of fixed HEK cells expressing KCNQ1 and FLAG-KCNE1 after sustained cPKC stimulation (1 μM cPKC activator peptide KAC1-1, 90 min). Nuclei were labeled with DAPI (in blue). Right, Summary data of normalized KCNE1 membrane localization. (B) Left, Representative Western Blots of whole-cell extracts obtained after sustained cPKC activation (1 μM cPKC activator peptide KAC1-1, 90 min). UNT. was untransfected sample. Data were normalized to GAPDH loading control expression. Right, Average of KCNE1/GAPDH expression ratio in 3 experiments. Control = control peptide C1. Scale bars, 5 μm. *p<0.05, (n = number of cells). (DOCX) [file pone.0237591.s004.docx]

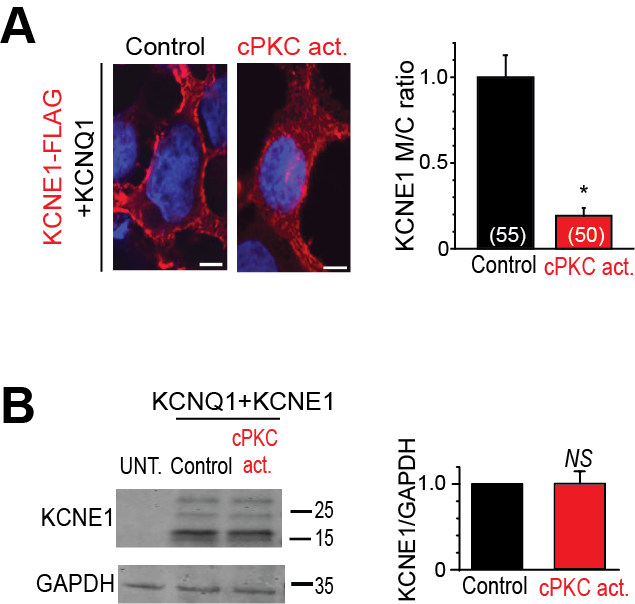


**Figure S4. The membrane localization of KCNE1 subunits is decreased in response to sustained cPKC stimulation without change of overall subunit expression.** **(A)** *Left*, Representative immunofluorescent images of fixed HEK cells expressing KCNQ1 and FLAG-KCNE1 after sustained cPKC stimulation (1 µM cPKC activator peptide KAC1-1, 90 min). Nuclei were labeled with DAPI (in blue). *Right,* Summary data of normalized KCNE1 membrane localization. **(B)** *Left,* Representative Western Blots of whole-cell extracts obtained after sustained cPKC activation (1 µM cPKC activator peptide KAC1-1, 90 min). UNT. was untransfected sample. Data were normalized to GAPDH loading control expression. *Right*, Average of KCNE1/GAPDH expression ratio in 3 experiments. Control =control peptide C1. Scale bars, 5 µm. *p<0.05, (n= number of cells).
