# Supplementary material for: The auxiliary subunit KCNE1 regulates KCNQ1 channel response to sustained calcium-dependent PKC activation
Source: PLoS One. 2020 Aug 24;15(8):e0237591. doi: 10.1371/journal.pone.0237591 (PMC7446858; doi:10.1371/journal.pone.0237591)
Supplement: S5 Fig — Western Blots of whole-cell extracts obtained after sustained cPKC activation (1 μM cPKC activator peptide KAC1-1, 90 min). UNT. marks untransfected samples. (DOCX) [file pone.0237591.s005.docx]

**
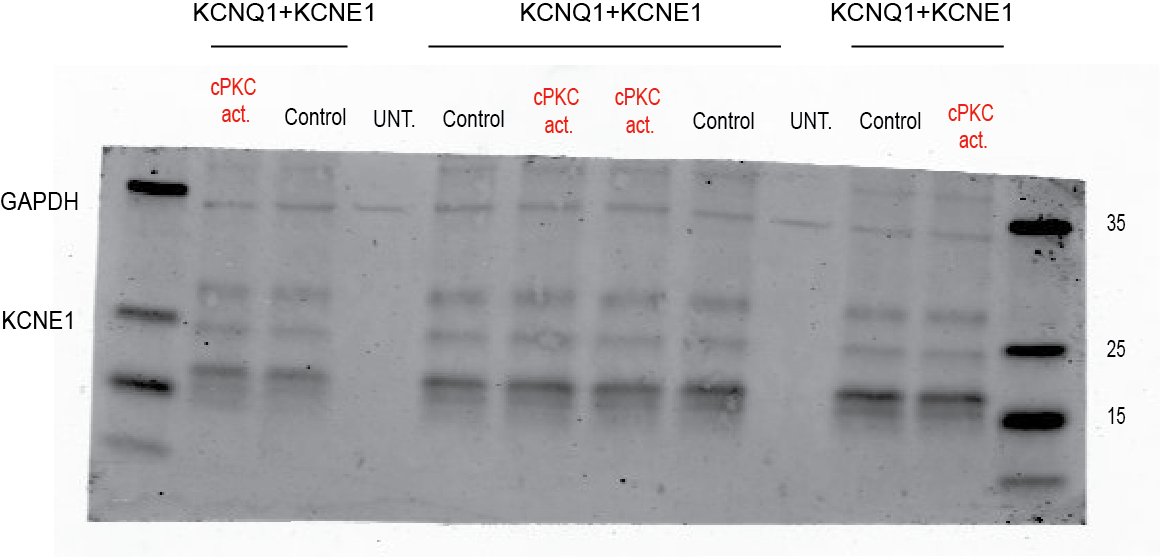
**

**Figure S5. Western Blots of whole-cell extracts obtained after sustained cPKC activation.** Western Blots of whole-cell extracts obtained after sustained cPKC activation (1 µM cPKC activator peptide KAC1-1, 90 min). UNT. marks untransfected samples.
