# Supplementary material for: The auxiliary subunit KCNE1 regulates KCNQ1 channel response to sustained calcium-dependent PKC activation
Source: PLoS One. 2020 Aug 24;15(8):e0237591. doi: 10.1371/journal.pone.0237591 (PMC7446858; doi:10.1371/journal.pone.0237591)
Supplement: S6 Fig — Average I-V plots (left) and V1/2 (right) of KCNQ1/KCNE1(S102A) channels at control condition (in black, control peptide C1) and sustained cPKC activation (1 μM cPKC activator peptide KAC1-1) for 90 min (in red). The solid lines in the left panels are Boltzman fits of experimental data. *p<0.05, n = the number of cells measured. (DOCX) [file pone.0237591.s006.docx]

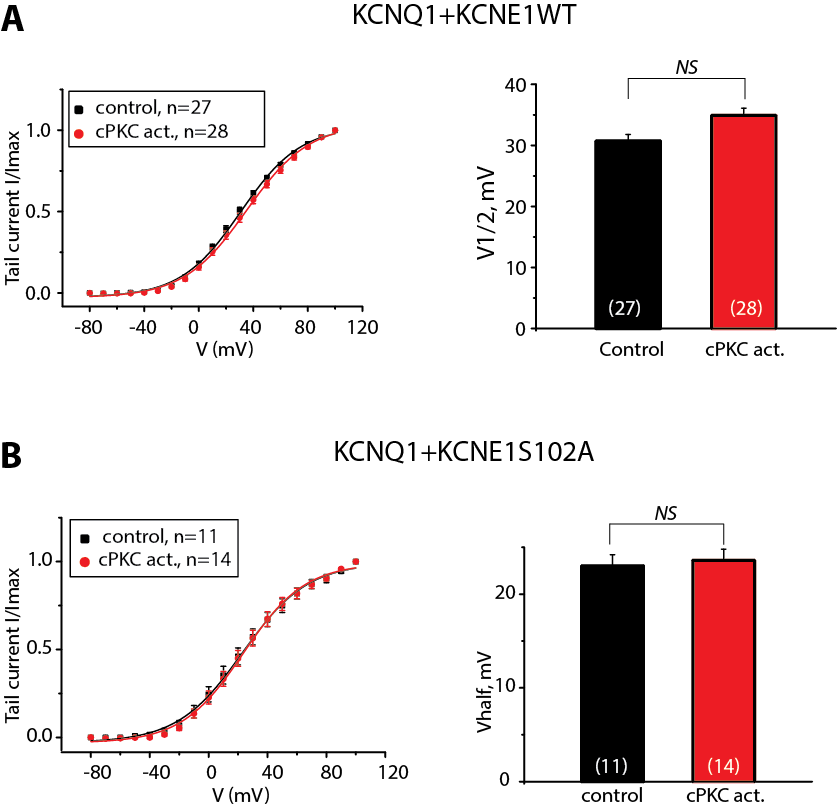


**Figure S6. Sustained cPKC activation did not change voltage dependence of KCNQ1/KCNE1(S102A) channel activation.** Average I-V plots (left) and V_1/2_ (right) of KCNQ1/KCNE1(S102A) channels at control condition (in black, control peptide C1) and sustained cPKC activation (1 µM cPKC activator peptide KAC1-1) for 90 min (in red). The solid lines in the left panels are Boltzman fits of experimental data. *p<0.05, n = the number of cells measured.
