# Supplementary material for: The auxiliary subunit KCNE1 regulates KCNQ1 channel response to sustained calcium-dependent PKC activation
Source: PLoS One. 2020 Aug 24;15(8):e0237591. doi: 10.1371/journal.pone.0237591 (PMC7446858; doi:10.1371/journal.pone.0237591)
Supplement: S7 Fig — Left, Representative images of cardiomyocytes expressing adenovirus of KCNQ1-GFP and KCNE1 at a ratio of 3:1, in the presence and absence of sustained α1-AR stimulation (Phe, 30 μM, 90 min), and cPKC inhibition (20 nM, LY333531, 90 min). Right, Summary data of membrane localization of KCNQ1 from experiments conducted as in the left panel. *p<0.05, n = number of cells measured. (DOCX) [file pone.0237591.s007.docx]

**
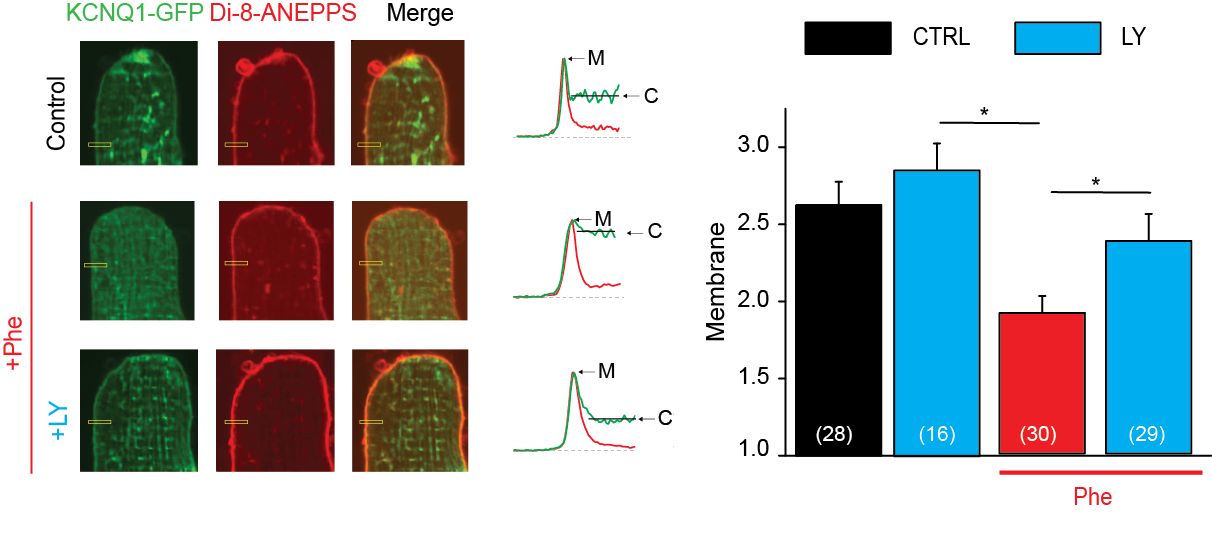
**

**Figure S7. Change in KCNQ1:KCNE1 stoichiometry does not affect cPKC-dependent IKs channel internalization in isolated adult rat ventricular myocytes.** *Left*, Representative images of cardiomyocytes expressing adenovirus of KCNQ1-GFP and KCNE1 at a ratio of 3:1, in the presence and absence of sustained α1-AR stimulation (Phe, 30 µM, 90 min), and cPKC inhibition (20 nM, LY333531, 90 min). *Right*, Summary data of membrane localization of KCNQ1 from experiments conducted as in the left panel. *p<0.05, n = number of cells measured
