# Supplementary material for: The auxiliary subunit KCNE1 regulates KCNQ1 channel response to sustained calcium-dependent PKC activation
Source: PLoS One. 2020 Aug 24;15(8):e0237591. doi: 10.1371/journal.pone.0237591 (PMC7446858; doi:10.1371/journal.pone.0237591)
Supplement: S8 Fig — (A) Left: representative confocal images of HEK cells expressing KCNQ1-GFP and the KCNE1 subunit, in the presence and absence of dorsomorphine (90 min). Cytoplasmic fluorescence was measured at the indicated region. Right: Summary data of KCNQ1 normalized membrane localization in experiments conducted as in the left panels. (B) Top: representative confocal images of HEK cells expressing KCNQ1-GFP and KCNE1 treated with Phe (30uM, 90min) in the presence and absence of dorsomorphine (90 min). Bottom: Summary data of KCNQ1-GFP cytoplasmic fluorescence in experiments conducted as in the top panels for cells treated with either Phe (30uM, 90min) or cPKCact (1uM, 90min) as indicated. For all conditions, control is significantly different from treated cells. Scale bars, 5 μm. *p<0.05. (n = cell number). (DOCX) [file pone.0237591.s008.docx]

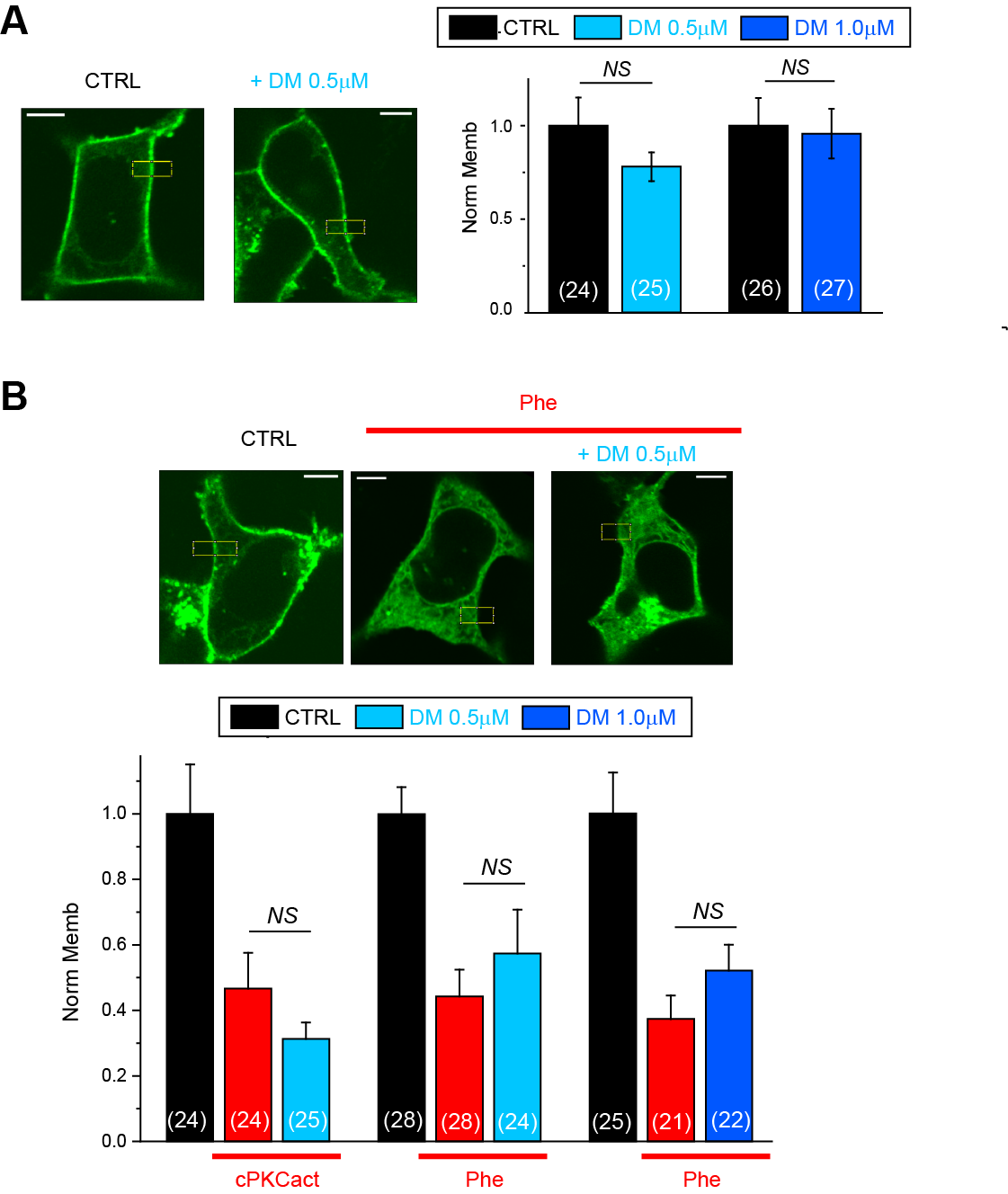


**Figure S8. AMPK inhibitor does not affect KCNQ1 membrane localization and cPKC mediated internalization.**

1. **Left:** representative confocal images of HEK cells expressing KCNQ1-GFP and the KCNE1 subunit, in the presence and absence of dorsomorphine (90 min). Cytoplasmic fluorescence was measured at the indicated region. **Right:** Summary data of KCNQ1 normalized membrane localization in experiments conducted as in the left panels. **(B)** **Top:** representative confocal images of HEK cells expressing KCNQ1-GFP and KCNE1 treated with Phe (30uM, 90min) in the presence and absence of dorsomorphine (90 min). **Bottom:** Summary data of KCNQ1-GFP cytoplasmic fluorescence in experiments conducted as in the top panels for cells treated with either Phe (30uM, 90min) or cPKCact (1uM, 90min) as indicated. For all conditions, control is significantly different from treated cells. Scale bars, 5 µm. *p<0.05. (n = cell number.)
