# Supplementary material for: The auxiliary subunit KCNE1 regulates KCNQ1 channel response to sustained calcium-dependent PKC activation
Source: PLoS One. 2020 Aug 24;15(8):e0237591. doi: 10.1371/journal.pone.0237591 (PMC7446858; doi:10.1371/journal.pone.0237591)
Supplement: S1 Raw images — (PDF) [file pone.0237591.s009.pdf]

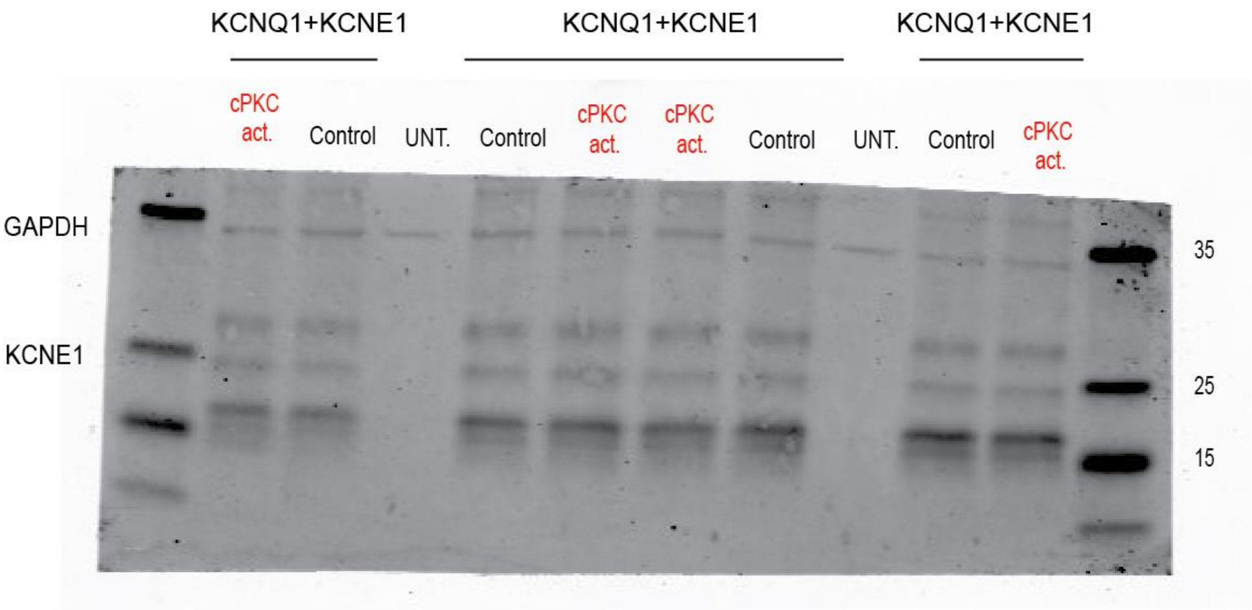


**Western Blots of whole-cell extracts obtained after sustained cPKC activation. (A)** Western Blots of whole-cell extracts obtained after sustained cPKC activation (1 µM cPKC activator peptide KAC1-1, 90 min). UNT. marks untransfected samples.
